# Neurotensin Expression in the Posterior Hypothalamus Is Influenced by an Increase in Kisspeptin Levels: Insights From In Vivo Rat Models and Hypothalamic Cell Models

**DOI:** 10.1155/ije/6698205

**Published:** 2025-07-31

**Authors:** Batjargal Lkhagvajav, Haruhiko Kanasaki, Tuvshintugs Tumurbaatar, Zhuoma Cairang, Aki Oride, Hiroe Okada, Satoru Kyo

**Affiliations:** Department of Obstetrics and Gynecology, Faculty of Medicine, Shimane University, Izumo 693-8501, Japan

**Keywords:** HPG axis, hypothalamus, kisspeptin, neurotensin

## Abstract

Neurotensin (NT) is a hypothalamic peptide that acts as a neurohormone and exerts a potent vascular effect. NT is also implicated in regulating the reproductive system. In the present study, we examined the role of NT in the hypothalamic–pituitary–gonadal axis using rat and cell-based models. In female rats, NT gene expression within the posterior part of the hypothalamus was increased by 1.53 ± 0.2-fold by ovariectomy (OVX), and this increase was prevented by estradiol (E2) supplementation after OVX. E2 administration to ovary-intact rats reduced NT gene expression in this part of the hypothalamus. Progesterone (P4) and dihydrotestosterone (DHT) administration also prevented the OVX-induced increase of NT gene expression, while P4 and DHT administration to ovary-intact rats reduced hypothalamic NT gene expression. As previously reported, Kiss-1 gene expression in the posterior part of the hypothalamus was increased by OVX, and this increase was repressed by sex steroid supplementation after OVX. In experiments using hypothalamic cell models, E2 slightly increased NT mRNA expression by 1.67 ± 0.19-fold in GT1-7 cells but failed to increase its expression in rHypoE-8 cells. E2 stimulation slightly increased Kiss-1 gene expression in GT1-7 and rHypoE8 hypothalamic cells by 1.45 ± 0.03-fold and 1.57 ± 0.25-fold, respectively. NT stimulation failed to increase Kiss-1 gene expression in GT1-7 cells but increased its expression significantly in rHypoE8 cells by 1.96 ± 0.22-fold. In contrast, kisspeptin significantly increased NT mRNA expression in GT1-7 (1.85 ± 0.18-fold) and rHypoE8 (4.41 ± 1.03-fold) cells. In the mHypoA-55 hypothalamic KNDy neuron cell model, kisspeptin also significantly increased NT gene expression by 2.13 ± 0.28-fold. Although E2 had some effect on NT expression in hypothalamic cells, kisspeptin significantly increased NT gene expression in three different hypothalamic cell lines. Given that the NT and Kiss-1 gene expression patterns in OVX rats and the effects of sex steroid supplementation were similar, the OVX-induced increase in NT gene expression in the hypothalamus might depend on the increase in Kiss-1/kisspeptin expression within this part of the hypothalamus.

## 1. Introduction

The female reproductive system is maintained by the hypothalamic–pituitary–gonadal (HPG) axis, and it is widely accepted that neurons expressing kisspeptin (encoded by the Kiss-1 gene) in the hypothalamus are positioned at the highest level of the HPG axis and govern the downstream endocrine systems. Briefly, kisspeptin-expressing neurons (Kiss-1 neurons) located in two different hypothalamic areas govern the release of gonadotropin-releasing hormone (GnRH) from GnRH neurons. In rodents, Kiss-1 neurons in the anteroventral periventricular nucleus (AVPV) region in the hypothalamus are involved in the surge secretion of GnRH, which induces a luteinizing hormone (LH) surge to evoke ovulation after oocyte maturation with the concomitant elevation of estradiol (E2). In contrast, Kiss-1 neurons in the arcuate nucleus (ARC) region in the hypothalamus control the basal pulsatile release of GnRH. Thus, Kiss-1 neurons in the AVPV are believed to be central for the E2-induced LH surge (positive feedback), while those in the ARC are believed to be central for the E2-induced negative feedback mechanism [1, 2]. This is evidenced by the fact that E2 increases Kiss-1 gene expression in the AVPV, whereas E2 represses its expression in the ARC [3, 4].

Kiss-1 neurons in the ARC coexpress kisspeptin, neurokinin B (NKB), and dynorphin (Dyn), which are referred to as KNDy neurons [5]. KNDy neurons play a role as a GnRH pulse generator by generating synchronized oscillatory patterns of activity after receiving auto-synaptic excitatory and inhibitory signals from NKB and Dyn, respectively [6]. However, it remains largely unknown whether KNDy neurons themselves are the essence of the GnRH pulse generator or whether other neuronal factors influence KNDy neurons.

Neurotensin (NT) is a neuropeptide that has potent vascular effects and was first isolated from the bovine hypothalamus almost 50 years ago [7]. NT is abundantly expressed in the central nervous system, and its expression is particularly high in the hypothalamus, including the AVPV and ARC, and can act as a neurotransmitter and neurohormone [7, 8]. NT is one of the factors that regulate pituitary hormone secretion and is involved in the secretion of growth hormones, adrenocorticotropic hormone, and prolactin [9–11]. NT also plays some roles in the regulation of the HPG axis by modulating gonadotropin secretion [12–14].

The mHypoA-55 hypothalamic cell model was a clonal cell line, determined to be ARC of female murine hypothalamic cells. mHypoA-55 cells express kisspeptin as well as estrogen receptors. These cells also express NKB and Dyn, suggesting that they could be used as a model for KNDy neurons [15]. We previously reported that NT is expressed in mHypoA-55 cells and is upregulated by E2 stimulation [16]. Furthermore, we found that exogenous NT stimulation reduces endogenous Kiss-1 gene expression in these cells [16]. From these observations, we speculated that NT could mediate the E2 signals to Kiss-1 neurons and affect the HPG axis. In the present study, we investigated the role of NT in the HPG axis in more detail. Because ovariectomy (OVX) has a huge impact on the HPG axis by depleting circulating E2, we examined its effect on the expression of NT in the rat hypothalamus. Furthermore, given that kisspeptin neurons are known to principally govern the HPG axis by regulating GnRH secretion, we investigated the relation between kisspeptin neurons and NT by examining the reciprocal effects of kisspeptin and NT expression, using three different hypothalamic cell models—GT1-7, rHypoE-8, and mHypoA-55 cells—all of which express both Kiss-1 and NT.

## 2. Materials and Methods

### 2.1. Materials

The following chemicals and reagents were obtained from the indicated sources: fetal bovine serum (Invitrogen, Carlsbad, CA); Dulbecco's modified Eagle's medium, penicillin–streptomycin, water-soluble E2, water-soluble progesterone (P4), and 5α-dihydrotestosterone (DHT) (Sigma-Aldrich Co., St. Louis, MO); NT (Life Technologies Japan, Ltd., Tokyo, Japan); kisspeptin (AnaSpec, Fremont, CA); and selective MEK inhibitor U0126 (Abcam, Cambridge, UK).

### 2.2. Animal Experiments

Six-week-old female Wistar rats were maintained under a 12-h light/dark cycle at 20°C–25°C with food (CE-2; CLEA Japan, Tokyo, Japan) and water available *ad libitum*. The rats received OVX under intraperitoneal anesthesia with an injection of medetomidine (0.15 mg/kg), midazolam (2 mg/kg), and butorphanol (2.5 mg/kg). Sham-operated (ovary-intact) rats were used as a control. After OVX, a pellet containing 0.25 mg E2 or 50 mg P4 (Innovative Research of America, Sarasota, FL) was implanted subcutaneously. The rats were housed for another 7 days and then euthanized under isoflurane anesthesia. For DHT administration, the rats received a daily subcutaneous injection of 25 mg/kg DHT in 140 μL sesame oil (Fujifilm Wako Pure Chemical Corp., Osaka, Japan). In the experiments using ovary-intact rats, sex steroids were administered for 7 days without OVX, and control rats were injected with 140 μL of sesame oil daily. The posterior area of the hypothalamus containing the ARC was removed for use in the experiments. This protocol was approved by the Ethics Committee of the Experimental Animal Center for Integrated Research at Shimane University (IZ31-51).

### 2.3. Cell Culture

The mouse GT1-7 GnRH-producing hypothalamic cell line was kindly provided by Dr. P. Mellon (University of California, San Diego, CA). The rHypoE-8 R8 embryonic rat hypothalamic cell line was purchased from COSMO Bio Co., Ltd. (Tokyo, Japan). The mHypoA-55 ARC-derived hypothalamic KNDy neuron model was purchased from Cedarlane Laboratories (Ontario, Canada). The cells were plated in 35-mm tissue culture dishes and incubated with high-glucose Dulbecco's modified Eagle's medium containing 10% heat-inactivated fetal bovine serum and 1% penicillin–streptomycin at 37°C under a humidified atmosphere of 5% CO_2_ in air. After 48 h, the culture medium was changed to high-glucose Dulbecco's modified Eagle's medium containing 1% heat-inactivated fetal bovine serum and 1% penicillin–streptomycin and incubated without (control) or with the test reagents for the indicated periods.

### 2.4. RNA Preparation, Reverse Transcription, PCR, and Quantitative Real-Time (RT) PCR

Total RNA was extracted from the posterior part of the rat hypothalamus, rHypoE-8, GT1-7, and mHypoA-55 cells using TRIzol-LS (Molecular Research Center, Inc., Cincinnati, OH). To obtain cDNA, 1.0 μg of total RNA was reverse-transcribed using an oligo-dT primer (Promega, Madison, WI) and prepared using a First-Strand cDNA Synthesis Kit (Invitrogen) and reverse transcription buffer. The preparation was supplemented with 10 mM dithiothreitol, 1 mM of each dNTP, and 200 U RNase inhibitor/human placenta ribonuclease inhibitor (#2310; Takara, Tokyo, Japan) in a final volume of 10 μL. The reaction was incubated at 37°C for 60 min. Quantification of NT and Kiss-1 gene expression was performed by quantitative RT-PCR (Takara TP900; Takara-Bio, Tokyo, Japan) using Universal ProbeLibrary Probes and FastStart Master Mix (Roche Diagnostics, Mannheim, Germany). The PCR primers were designed based on the published sequences of NT and Kiss-1 [16] as follows: NT, forward 5′-GTGTGGACCTGCTTGTCAGA-3′ and reverse 5′-TCATGCATGTCTCCTGCTTC-3′; and Kiss-1, forward 5′-ATGATCTCGCTGGCTTCTTGG-3′ and reverse 5′-GGTTCACCACAGGTGCCATTTT-3′. The simultaneous measurement of target mRNAs and GAPDH permitted the normalization of transcript levels. Each set of primers included a no-template control. The thermal cycling conditions were as follows: 10 min of denaturation at 94°C, followed by 40 cycles of 94°C for 15 s and 55°C for 1 min. Reactions were followed by melting curve analysis (55°C–95°C). To determine PCR efficiency, 10-fold serial dilutions of cDNA were used as described previously [17]. PCR conditions were optimized to obtain > 95% efficiency, and only those reactions with efficiencies between 95% and 105% were included in subsequent analyses. Relative differences in cDNA concentration between baseline and experimental conditions were calculated using the comparative threshold cycle (Ct) method [18]. Briefly, for each sample, ΔCt was calculated for normalization against the internal control using the following equation: ΔCt = Ct (gene) − Ct (GAPDH). To obtain differences between experimental and control conditions, ΔΔCt was calculated as ΔCt (sample) − ΔCt (control). Relative mRNA levels were calculated using the following equation: fold difference = 2ΔΔCt.

### 2.5. Western Blot Analysis

Cell extracts were lysed on ice with radioimmunoprecipitation assay buffer (phosphate-buffered saline, 1% NP-40, 0.5% sodium deoxycholate, and 0.1% sodium dodecyl sulfate) containing 0.1 mg/mL phenylmethyl sulfonyl fluoride, 30 mg/mL aprotinin, and 1 mM sodium orthovanadate, scraped for 20 s, and centrifuged at 14000 × *g* for 10 min at 4°C. The protein concentration in cell lysates was measured using the Bradford method. Denatured protein (15 μg per well) was resolved by 12% sodium dodecyl sulfate–polyacrylamide gel electrophoresis according to standard protocols and transferred onto polyvinylidene difluoride membranes (Hybond-P PVDF; Amersham Biosciences, Little Chalfont, UK). Membranes were incubated with anti-kisspeptin (1:500 dilution; Abcam, Cambridge, UK) and anti-NT (1:200 dilution; Santa Cruz Biotechnology, Inc., Dallas, TX) antibodies in Blotto overnight at 4°C and washed three times for 10 min per wash with Tris-buffered saline/1% Tween. Subsequent incubation with horseradish peroxidase-conjugated antibodies was performed for 1 h at room temperature in Blotto, and additional washes were performed appropriately. Following enhanced chemiluminescence detection (Amersham Biosciences), membranes were exposed to X-ray film (Fujifilm, Tokyo, Japan). Fetal rat brain tissue was used as a positive control, and this experimental protocol was approved by the Animal Care and Use Committee of the Experimental Animal Center for Integrated Research at Shimane University (IZ27-82).

### 2.6. Statistical Analysis

All experiments were repeated independently at least three times. Each experiment in each experimental group was performed using duplicate samples (quantitative RT-PCR). When mRNA expression was determined, two samples were assayed in duplicate. From four sets of data, the mean ± standard error of the mean (SEM) was calculated. Using three means from three independent experiments, data were analyzed statistically and the mean ± SEM was calculated. Statistical analysis was performed using Student's *t*-test in the experiments comparing two stimulation groups. One-way analysis of variance with Bonferroni's *post hoc* test was conducted to analyze the experiments that determined the effects of two doses of stimulant on target gene expression. Statistical significance was assessed at a threshold of *p* < 0.05. All analyses were performed using Prism 6.07 Software (GraphPad Software, San Diego, CA).

## 3. Results

### 3.1. Effects of OVX and E2 Supplementation on NT mRNA Expression in the Posterior Part of the Hypothalamus and Effects of E2 Administration on Ovary-Intact Rats

**Figure 1:**
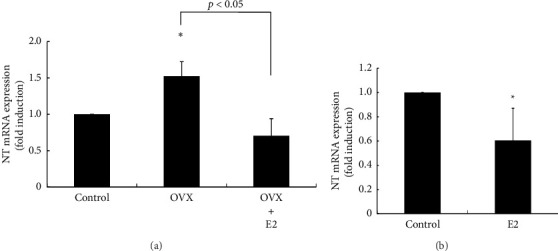
Effects of OVX and E2 supplementation on NT gene expression in the posterior part of the hypothalamus. (a) Six-week-old rats received OVX or a 0.25-mg E2 pellet was implanted subcutaneously after OVX (OVX + E2). Sham-operated (ovary-intact) rats were used as a control. (b) A 0.25-mg E2 pellet was implanted subcutaneously in ovary-intact six-week-old rats. Sham-operated (no E2 pellet) rats were used as a control. Seven days later, the rats were euthanized, and the posterior part of the hypothalamus was removed. mRNA was extracted from the hypothalamic tissues and reverse-transcribed. NT mRNA levels were measured by quantitative RT-PCR. Samples for each experimental group were run in duplicate and normalized to the mRNA levels of GAPDH as a housekeeping gene. The results are expressed as fold induction over control and presented as the mean ± SEM. ^∗^*p* < 0.05 versus control. The difference between OVX and OVX + E2 rats was significant (*p* < 0.05).

To examine the effects of OVX on NT gene expression in the hypothalamus, rats were ovariectomized and NT mRNA expression was determined using the posterior part of the hypothalamus because Kiss-1 neurons (KNDy neurons) in the ARC region of the hypothalamus, which maintain the basal secretion of GnRH, are present in this area. Following OVX, NT mRNA expression within this hypothalamic area was significantly increased by 1.52 ± 0.20-fold compared with sham-operated control rats. E2 supplementation after OVX completely eliminated the OVX-induced increase in NT gene expression (Figure 1(a)). In ovary-intact rats, E2 administration reduced hypothalamic NT mRNA expression by 0.6 ± 0.26-fold compared with nontreated rats (Figure 1(b)). An OVX-induced increase in NT gene expression was not observed in the tissues from the anterior part of the hypothalamus, which includes the AVPV region (data not shown).

### 3.2. Effects of Sex Steroids on NT Gene Expression in Rats After OVX and in Ovary-Intact Rats

**Figure 2:**
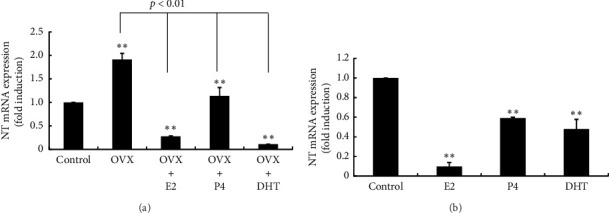
Effects of OVX and E2, P4, and DHT supplementation on NT gene expression in the posterior part of the hypothalamus. (a) Six-week-old rats received OVX. After OVX, a 0.25-mg E2 pellet (OVX + E2) or 50-mg P4 pellet (OVX + P4) was implanted subcutaneously, or the rats received a daily subcutaneous injection of 25 mg/kg DHT after OVX (OVX + DHT). Sham-operated (ovary-intact) rats were used as a control. (b) Seven-week-old ovary-intact rats were implanted with a pellet containing 0.25-mg E2 or 50-mg P4 or received a daily subcutaneous injection of 25 mg/kg DHT. Control rats were injected with 140 μL of sesame oil daily. Seven days later, the rats were euthanized, and the posterior part of the hypothalamus was removed. mRNA was extracted from the hypothalamic tissues and reverse-transcribed. NT mRNA levels were measured by quantitative RT-PCR. Samples for each experimental group were run in duplicate and normalized to the mRNA levels of GAPDH as a housekeeping gene. The results are expressed as fold induction over control and presented as the mean ± SEM. ^∗∗^*p* < 0.01, ^∗^*p* < 0.05 versus control. The difference between OVX and OVX + E2, OVX + P4, and OVX + DHT was significant (*p* < 0.01).

The OVX-induced increase in NT gene expression in the posterior hypothalamus was repressed by E2 supplementation after OVX. P4 supplementation also inhibited the increase in NT mRNA expression after OVX, but its inhibitory effect was limited compared with E2 supplementation. Similar to the effect of E2, DHT supplementation completely inhibited the OVX-induced increase in NT gene expression (Figure 2(a)). P4 and DHT administration also reduced NT mRNA expression in the posterior hypothalamus in ovary-intact rats, but their inhibitory effect on NT gene expression was modest compared with the effect of E2 administration (Figure 2(b)).

### 3.3. Effects of OVX and Sex Steroid Supplementation on Kiss-1 Gene Expression in the Posterior Part of the Hypothalamus

**Figure 3:**
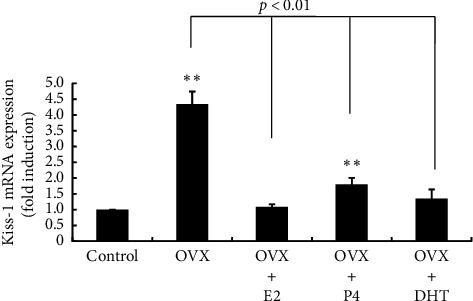
Effects of OVX and E2, P4, and DHT supplementation on Kiss-1 gene expression in the posterior part of the hypothalamus. Six-week-old rats received OVX. After OVX, a 0.25-mg E2 pellet (OVX + E2) or 50-mg P4 pellet (OVX + P4) was implanted subcutaneously, or the rats received a daily subcutaneous injection of 25 mg/kg DHT after OVX (OVX + DHT). Sham-operated (ovary-intact) rats were used as a control. Seven days later, the rats were euthanized, and the posterior part of the hypothalamus was removed. mRNA was extracted from the hypothalamic tissues and reverse-transcribed. Kiss-1 mRNA levels were measured by quantitative RT-PCR. Samples for each experimental group were run in duplicate and normalized to the mRNA levels of GAPDH as a housekeeping gene. The results are expressed as fold induction over control and presented as the mean ± SEM. ^∗∗^*p* < 0.01, ^∗^*p* < 0.05 versus. control. The difference between OVX and OVX + E2, OVX + P4, and OVX + DHT was significant (*p* < 0.01).

We confirmed the OVX-induced change in Kiss-1 gene expression. As reported previously, Kiss-1 gene expression in the area containing the ARC was significantly increased by 44.34 ± 0.4-fold following OVX compared with sham-operated control rats. The OVX-induced increase in Kiss-1 gene expression was almost completely repressed by E2, P4, and DHT supplementation after OVX (Figure 3).

### 3.4. Kiss-1 and NT Expression in Hypothalamic Cell Models

**Figure 4:**
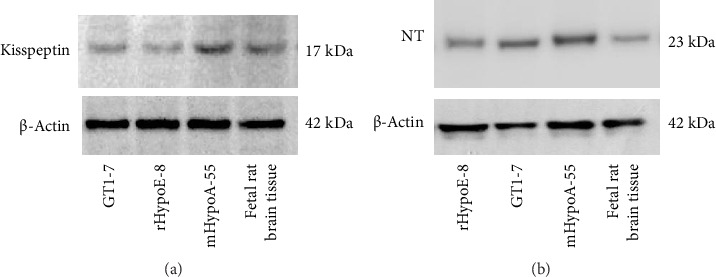
Cell lysates (10 μg protein) from GT1-7, rHypoE-8, and mHypoA-55 cells were analyzed by sodium dodecyl sulfate–polyacrylamide gel electrophoresis followed by immunoblotting and incubation with antibodies against kisspeptin (a) and NT (b). β-Actin was detected as an internal control. Proteins from fetal rat brain tissues were used as a positive control. The bands were visualized using a horseradish peroxidase-conjugated secondary antibody.

To investigate the relationship between NT and kisspeptin expression within the hypothalamus, hypothalamic cell lines were examined. GT1-7 cells originate from mouse GnRH neurons [19], and these cells also expressed kisspeptin (Figure 4(a)). Rat embryo hypothalamus-derived rHypoE-8 cells also expressed kisspeptin [20] (Figure 4(a)). In addition, the GT1-7 and rHypoE-8 hypothalamic cell models expressed NT at the protein level (Figure 4(b)). Therefore, we used GT1-7 and rHypoE-8 cells as hypothalamic cells that coexpress kisspeptin and NT. We also used the mHypoA-55 hypothalamic KNDy neuron cell model that originates from the ARC of the mouse hypothalamus, and this cell line expressed kisspeptin and NT at the protein level (Figures 4(a) and 4(b)) [15]. We have confirmed that, in addition to Kiss-1, mHypoA-55 KNDy neuron cells also express NKB and Dyn, whereas rHypoE-8 expresses NKB but not Dyn. GT1-7 cells did not express NKB or Dyn (data not shown).

### 3.5. Effects of E2 on NT and Kiss-1 Gene Expression in Hypothalamic Cell Models

**Figure 5:**
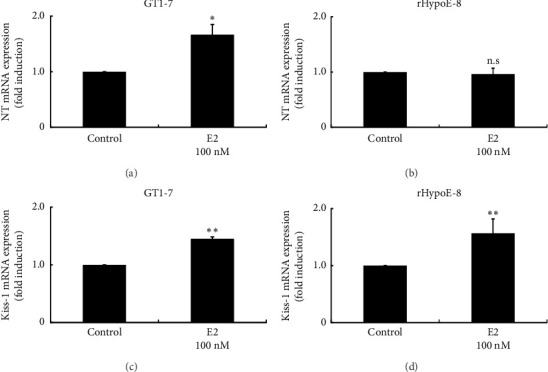
Effects of E2 on NT and Kiss-1 gene expression in hypothalamic cell models. GT1-7 (a, c) and rHypoE-8 (b, d) cells were stimulated with 100 nM E2 for 24 h, after which mRNA was extracted and reverse-transcribed. NT (a, b) and Kiss-1 (c, d) mRNA levels were measured by quantitative RT-PCR. Results are expressed as fold induction over unstimulated cells and presented as mean ± SEM values of three independent experiments, each performed with duplicate samples. ^∗∗^*p* < 0.01, ^∗^*p* < 0.05 versus control. n.s. = Not significantly different.

First, we examined the direct effects of E2 on NT gene expression in two hypothalamic cell models. In GT1-7 cells, 100 nM E2 stimulation slightly but significantly increased NT mRNA expression by 1.67 ± 0.19-fold (Figure 5(a)). In rHypoE-8 cells, the same concentration of E2 failed to increase NT mRNA expression (Figure 5(b)). E2 stimulation slightly increased Kiss-1 gene expression in GT1-7 cells by 1.45 ± 0.032-fold (Figure 5(c)). Similarly, E2 stimulation slightly but significantly increased Kiss-1 gene expression in rHypoE-8 cells by 1.57 ± 0.25-fold (Figure 5(d)). We have previously reported that E2 stimulation increased NT gene expression, while E2 stimulation for 24 h did not modify Kiss-1 gene expression in the KNDy neuron cell model mHypoA-55 cells [16].

### 3.6. Effects of NT on Kiss-1 Gene Expression and the Effects of Kisspeptin on NT Gene Expression in Hypothalamic Cell Models

**Figure 6:**
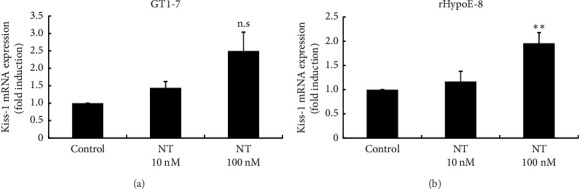
Effects of NT on Kiss-1 gene expression in hypothalamic cell models. GT1-7 (a) and rHypoE-8 (b) cells were stimulated with the indicated concentration of NT for 24 h, after which mRNA was extracted and reverse-transcribed. NT mRNA levels were measured by quantitative RT-PCR. Results are expressed as fold induction over unstimulated cells and presented as mean ± SEM values of three independent experiments, each performed with duplicate samples. ^∗^*p* < 0.05 versus control. n.s. = Not significantly different.

**Figure 7:**
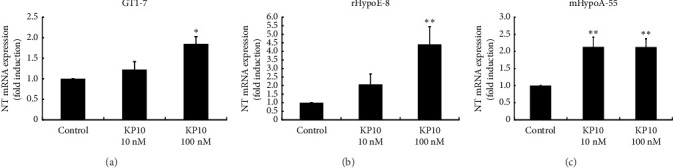
Effects of kisspeptin (KP10) on NT gene expression in hypothalamic cell models. GT1-7 (a), rHypoE-8 (b), and mHypoA-55 (c) cells were stimulated with the indicated concentration of KP10 for 24 h, after which mRNA was extracted and reverse-transcribed. NT mRNA levels were measured by quantitative RT-PCR. Results are expressed as fold induction over unstimulated cells and presented as mean ± SEM values of three independent experiments, each performed with duplicate samples. ^∗∗^*p* < 0.01, ^∗^*p* < 0.05 versus control.

Because the NT gene expression pattern in the OVX rat hypothalamus was similar to that of Kiss-1 gene expression in this region, we next examined the relationship between NT and kisspeptin using hypothalamic cells. Stimulation of GT1-7 cells with 100 nM NT increased Kiss-1 gene expression by 2.50 ± 0.54-fold, but this increase was not statistically significant (Figure 6(a)). However, in rHypoE-8 cells, 100 nM NT stimulation caused a small but significant increase in Kiss-1 gene expression by 1.96 ± 0.22-fold (Figure 6(b)). In contrast, 100 nM kisspeptin significantly increased NT mRNA expression in GT1-7 cells by 1.85 ± 0.18-fold compared with nonstimulated control cells (Figure 7(a)). Similarly, 100 nM kisspeptin significantly increased NT mRNA expression in rHypoE-8 cells by 4.41 ± 1.03-fold (Figure 7(b)). mHypoA-55 cells are used as a KNDy neuron cell model that originated from the ARC of the hypothalamus. Given that E2 represses Kiss-1 expression under certain conditions in these cells [15], they were used as a model for KNDy neurons. Similar to the phenomena observed in GT1-7 and rHypoE8 hypothalamic cells, kisspeptin significantly increased NT mRNA expression by 2.13 ± 0.28-fold at 10 nM and 100 nM (Figure 7(c)).

### 3.7. Effect of MEK Inhibitor, U0126, on KP10-Induced NT Gene Expression

**Figure 8:**
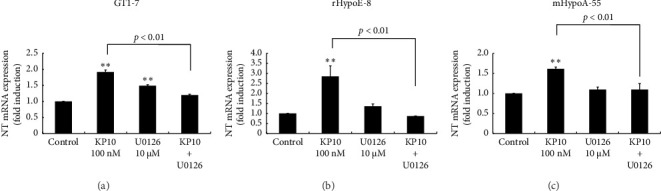
Effect of MEK inhibitor U0126 on NT gene expression in KP10-induced NT gene expression in hypothalamic cell models. GT1-7 (a), rHypoE-8 (b), and mHypoA-55 (c) cells were stimulated with 100 nM KP10 for 24 h in the absence or presence of 10 μM U0126. Prior to stimulation, cells were pretreated with U0126 for 1 h. Then, mRNA was extracted and reverse-transcribed. NT mRNA levels were measured by quantitative RT-PCR. Results are expressed as fold induction over unstimulated cells and presented as the mean ± SEM of three independent experiments, each performed with duplicate samples. ^∗∗^*p* < 0.01 versus control. The difference between KP10 and KP10 + U0126 was statistically significant (*p* < 0.01) (a–c).

Kisspeptin is known to bind mainly to Gq protein-coupled receptors [21]. Because KP10 strongly increases the extracellular signal-regulated kinase (ERK) signaling pathway in hypothalamic cells [22], we examined the possible involvement of ERK pathways in KP10-induced NT gene expression. When rHypoE-8, GT1-7, and mHypoA-55 cells were preincubated with 10 μM U0126 (a specific ERK inhibitor), the KP10-induced increase in NT gene expression was almost completely eliminated (Figure 8).

## 4. Discussion

We previously showed that NT expression is upregulated by E2 in the mHypoA-55 hypothalamic KNDy neuron cell line. We also reported that Kiss-1 gene expression is repressed in mHypoA-55 cells by NT stimulation [16]. Because KNDy neurons underlie the pulsatile secretion of GnRH and are thought to be a target for the negative feedback mechanism of sex steroids [4, 23], we speculated that NT-producing neuronal cells that respond to E2 could be positioned upstream of KNDy neurons and mediate the E2 signal to KNDy neurons. Because OVX induces the rapid depletion of circulating E2 and removes the negative feedback mechanism of E2, we expected that NT expression in the brain would be strongly influenced by OVX if NT expressed in the brain has some role in the HPG axis. As expected, NT gene expression in the posterior hypothalamus was significantly changed by OVX. We targeted the posterior part of the hypothalamus because it contains the ARC, where KNDy neurons are located, which play a pivotal role in E2-induced negative feedback mechanisms in the HPG axis. However, contrary to our expectations, OVX induced a significant increase in NT gene expression in this part of the hypothalamus. The OVX-induced increase in NT gene expression was completely inhibited by E2 supplementation after OVX, indicating that its expression depends on the depletion of E2. We had initially speculated that NT expression within this part of the hypothalamus would be suppressed by OVX *in vivo* because in our previous experiments using mHypoA-55 KNDy neuron cells, E2 increased NT gene expression [16]. But this opposite result in an animal model suggests that the depletion of E2 strongly stimulated NT expression in vivo and this is evidenced by the observation that E2 administration to ovary-intact rats reduced NT gene expression in this area of the hypothalamus. Therefore, it seems that E2 has a negative impact on NT gene expression in the posterior part of the hypothalamus *in vivo*.

The OVX-induced increase in NT gene expression in the hypothalamus is most likely a phenomenon associated with an increase in kisspeptin or Kiss-1 levels in this region because the changes in the gene expression pattern following OVX and the effect of supplementation with E2 or other sex steroids after OVX were exactly the same for the NT and Kiss-1 genes. The OVX-induced increase in Kiss-1 and NT gene expression was prevented by the administration of E2, P4, and androgen in female rats. This implied that androgen might also have a negative effect on the HPG axis. Furthermore, in three different Kiss-1-expressing hypothalamic models, GT1-7, rHypoE-8, and mHypoA-55 cells, kisspeptin stimulation significantly increased NT gene expression, suggesting that its expression in the hypothalamus could be upregulated by kisspeptin stimulation. Previously, Kiss-1 gene expression in the ARC of the hypothalamus was reported to be increased by OVX and decreased by E2 supplementation [4]. We also confirmed these phenomena in the present study. Thus, it seems reasonable to consider that locally expressed kisspeptin, which was increased by OVX, stimulates NT expression in these regions.

In the hypothalamic cell lines used in this study, E2 had a distinct effect on NT gene expression. E2 slightly but significantly increased NT gene expression in GT1-7 cells, but not in rHypoE-8 cells. In our previous experiments using the mHypoA-55 KNDy neuron cell model, E2 increased NT expression [16]. Because these hypothalamic cell lines exhibit heterogeneous characteristics, the response of NT gene expression to E2 stimulation varied in each cell model. However, even if E2 has some effect on NT expression in hypothalamic cells *in vivo*, its impact on NT expression seems to be very limited because E2 increased NT gene expression by less than 2-fold in GT1-7 and mHypoA-55 cells. Similarly, changes in Kiss-1 gene expression in response to E2 varied in a cell line-specific manner. E2 (24 h stimulation) increased Kiss-1 expression in GT1-7 cells by 1.45 ± 0.032-fold and in rHypoE-8 by 1.57 ± 0.25-fold. In mHypoA-55 KNDy neurons, stimulation with E2 for 24 h does not change Kiss-1 gene expression, although 4 h stimulation represses it [15, 16]. In addition, in the mHypoA-50 hypothalamic cell model, which was derived from the AVPV, E2 increases Kiss-1 gene expression [15, 24]. As for the response to E2 and Kiss-1 gene expression, it seems that rHypoE-8 and GT1-7 cells have a characteristic similar to that of mHypoA-50 AVPV cell models. However, rHypoE-8 expresses the gene for NKB, which is expressed in KNDy neurons but not in Dyn, whereas GT1-7 cells do not express NKB or Dyn. Therefore, our observations demonstrate the complexity and heterogeneity of the neuronal phenotypes of hypothalamic cells and also suggest that the response to E2 varies in a cell type–dependent manner.

Considering the observations that E2 did not have a significant impact on NT expression and did not modulate its expression in different hypothalamic cell models and that OVX strikingly increased NT gene expression in the ARC of the hypothalamus and its increase was prevented by E2, it is natural to think that NT expression within the hypothalamus is not principally controlled by E2. In contrast, NT stimulation decreased Kiss-1 gene expression in the mHypoA-55 KNDy neuron cell model [16], while it slightly increased Kiss-1 expression in rHypoE-8 cells, but not in GT1-7 cells, suggesting that the increase in Kiss-1 gene expression in the posterior part of the hypothalamus, which contains the ARC, was not induced by the increase in NT after OVX *in vivo*.

We speculated that kisspeptin could increase NT expression within the posterior part of the hypothalamus. This was evidenced by the observation that the pattern of NT gene expression in OVX with or without sex steroid supplementation was identical to the pattern of Kiss-1 gene expression in the posterior area of the hypothalamus. In addition, three different Kiss-1-expressing hypothalamic cell lines responded to kisspeptin and increased NT gene expression *in vitro*. The KP10-induced increase in NT gene expression might depend on the KP10-induced ERK signaling pathways because inhibition of the ERK pathways significantly inhibited NT gene expression in all three hypothalamic cell lines (GT1-7, rHypoE-8, and mHypoA-55). The promoter region of the NT gene contains a cAMP response element (CRE)/activator protein 1 (AP1)-like element that binds both AP1 and CRE-binding protein [25]. Because these transcription factors were activated by ERK signaling pathways [26, 27], they might be involved in KP10-induced NT gene expression in hypothalamic cells. Indeed, similar expression patterns of Kiss-1 and NT were observed in another part of the hypothalamus; NT gene expression in the medial preoptic nucleus of the hypothalamus is temporally increased during a GnRH/LH surge [28, 29], when Kiss-1 expression is increased in the AVPV of the hypothalamus [4]. However, it is obvious that NT does not function solely under the control of kisspeptin-expressing neurons. The direct administration of NT into the medial optic area evokes LH secretion in OVX rats [30], while the injection of antiserum against NT into this area blocks the surge secretion of LH [31]. These observations indicate that NT itself has the ability to modulate GnRH or gonadotropin secretion without the influence of E2 or kisspeptin. A more recent study by Dungan Lemko et al. demonstrated that NT expression was increased by E2 in the AVPV region of the female murine hypothalamus, but they did not observe an increase in circulating levels of LH after injection of NT into the cerebral ventricles [32]. In addition, it is also obvious that NT has a direct effect on pituitary adrenocorticotropic hormone and prolactin secretion [9, 11].

## 5. Conclusion

In this study, we found that the NT and Kiss-1 gene expression patterns after OVX were identical in the posterior hypothalamus, which contains the ARC. The NT gene expression pattern in this region after sex steroid supplementation in OVX or ovary-intact rats was identical to that observed previously for Kiss-1 gene expression in this area. Considering the fact that kisspeptin significantly increased NT expression in several hypothalamic neuronal cell lines, it is plausible to think that NT-producing neurons in this area are under the strong influence of kisspeptin-producing neurons. We speculate that this phenomenon is not likely to be common to all brain areas where NT is expressed. However, our present observation did not directly prove that the OVX-induced increase in NT is actually due to the increase in kisspeptin within the hypothalamus. To prove this scenario, kisspeptin receptor antagonist should be administered after OVX, and NT expression should be examined in the thalamus. Alternatively, the effect of direct administration of kisspeptin into the hypothalamus should be considered to ascertain the direct effect of kisspeptin on NT expression. In addition, we need to clarify the expression levels of the receptor for kisspeptin and NT in each of the hypothalamic cell models. Nevertheless, our present results provide some information for understanding the function of NT in the vicinity of Kiss-1 neurons in this area.

## Data Availability

The datasets used and/or analyzed during this study are available from the corresponding author upon reasonable request.
